# Alcohol’s Interactions With Circadian Rhythms: A Focus on Body Temperature

**Published:** 2001

**Authors:** Jill A. Wasielewski, Frank A. Holloway

**Affiliations:** Jill A. Wasielewski, Ph.D., is an associate study director at MPI Research, Mattawan, Michigan. Frank A. Holloway, Ph.D., is the director of the biological psychology program in the Department of Psychiatry and Behavioral Sciences, University of Oklahoma Health Sciences Center, Oklahoma City, Oklahoma

**Keywords:** circadian rhythm, body temperature, thermoregulation, hyperthermia, hypothermia, physiological AODE (effects of alcohol or other drug [AOD] use, abuse, and dependence), pharmacokinetics, time of day, AOD sensitivity, AOD preference

## Abstract

The complex interaction between alcohol and the body’s circadian rhythm has become a rapidly expanding area in chronopharmacology. This area has key implications for the field of alcohol research, because understanding alcohol’s effects on the body’s internal clock will aid scientists in designing medications and behavioral interventions for treating alcohol abuse and dependence. A number of studies provide evidence that alcohol sensitivity and preference vary with circadian timing. However, only a few studies support alcohol’s ability to influence the circadian phase directly. This review focuses on studies examining how alcohol and the body’s circadian rhythm interact, using body temperature as an index of circadian rhythm function. Though the research is limited, findings indicate that alcohol sensitivity and preference for drinking do indeed appear to vary with circadian timing and that alcohol may act directly on the central pacemaker to alter circadian functioning.

The complex interaction between alcohol and the body’s internal circadian rhythm (CR) or clock is a rapidly expanding area of research. The importance of the interaction between drugs, including alcohol, and CR is initially evident in the temporal, or time-related, restraints on experimentation. Pharmacological studies usually are performed at the same time each day to control for the profound effects that CR has over the mammalian system. Furthermore, this concept often is carried over into clinical administration, because many physicians recommend taking certain drugs at a specific time of day.

When the circadian effect is applied specifically to alcohol, however, other implications arise. For instance, recent studies have likened an alcohol hangover to jet-lag-like circadian disruption (i.e., phase shifts) of the body’s normal rhythm (Gauvin et al. 1997*b*). This internal jet lag is known to promote alcohol consumption directly by causing these phase shifts in the body’s internal clock (Gauvin et al. 1997*a*) and could potentially promote drinking indirectly through a disruption of CR caused by alcohol consumption (Holloway et al. 1993).

This review focuses on several variants of the alcohol and CR interaction using body temperature (T_b_) as an index of CR output. T_b_ varies throughout the day in a relatively stable and predictable rhythm. Therefore, examining changes in the body’s temperature rhythm, which, in people, peaks during late afternoon and reaches its lowest point during early morning, offers scientists a way of assessing the circadian system. Body temperature has a unique and reciprocal relationship with alcohol and the mammalian CR in that a change in one of these components often will lead to changes in another. Furthermore, alcohol and CR have the ability to interact with temperature at both the cellular and behavioral levels. It is important to note that both CR and alcohol simultaneously impose a myriad of behavioral and physiological effects on T_b_. With this in mind, temperature serves as a fitting, yet dynamic, variable on which to concentrate.

This article begins with a summary of basic chronopharmacology as it relates to alcohol, followed by an analysis of the relationship between alcohol and body temperature. The physiological and behavioral effects of alcohol as a function of time of day also are explored, followed by a discussion of how alcohol exerts an influence over the CR of T_b_. These distinct, yet related, objectives reflect the need to better integrate the alcohol and CR fields.

In addition to the association between increased alcohol consumption and CR alteration, alcohol-induced CR disruption could potentially diminish an individual’s ability to perform at optimum level. At least three interacting rhythmic effects exist: the CRs of (1) performance efficiency, (2) alcohol consumption, and (3) alcohol’s effects on performance efficiency.

Depending on the level of responsibility of the person (e.g., an air traffic controller or a truck driver), such circadian effects could pose a serious danger to both the affected person and other people. Therefore, a clearer understanding of how the circadian system impacts alcohol consumption and vice versa will most likely provide a foundation for pharmacological and behavioral advances in the treatment of alcohol abuse and addiction as well as assist in solving problems related to public safety.

## Basic Chronopharmacology of Alcohol: Chronokinetics, Chronesthesy, and Chronergy

This section of the article compiles a variety of CR-related definitions and explanations adapted from reviews by Reinberg (1992) and Bruguerolle (1994). This short synopsis is intended to provide a foundation for discussion of the various topics related to this field of study.

**Figure 1:**
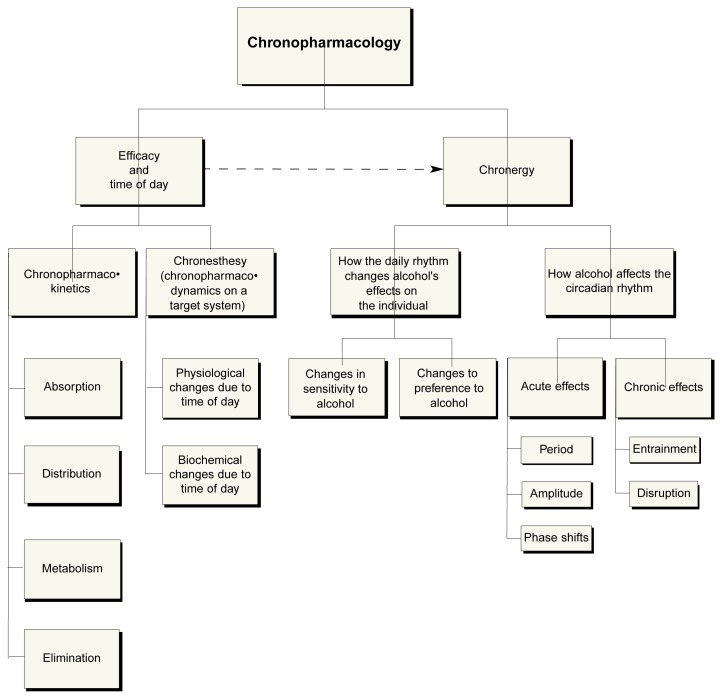
A representation of the field of chronopharmacology as it relates to alcohol.

The chronopharmacology of alcohol can be divided into two main areas (see figure 1). The first area focuses on how alcohol’s effects (i.e., its efficacy) are modified according to the time of day at which the alcohol is administered—that is, how alcohol interacts with the body’s physiological components at a particular time of day. The second area of focus, chronergy, takes a broader approach, beyond simple time-of-day effects, to determine the influence of a drug on the individual as a whole. These two areas of focus address the complex functional interactions between the organism’s CR and how it is influenced by or influences the body’s behavioral response to alcohol.

### Efficacy and Time of Day

#### Chronopharmacokinetics

Efficacy and time of day can be classified into chronopharmacokinetics and chronesthesy. The field of chronopharmacokinetics, similar to pharmacokinetics, investigates characteristic properties of the (1) absorption, (2) distribution, (3) metabolism, and (4) elimination of a particular substance related to a specific time course.

Absorption, or the rate and extent to which a drug (such as alcohol) disperses to the bloodstream, can be determined by a number of factors (e.g., pH levels and the rate of blood flow), all of which may play a part in chronopharmacology. These factors could be implicated in the variation observed in temporal drug effects. The distribution of alcohol within the body also can vary according to time. For instance, the degree to which the drug binds to molecules in the bloodstream (i.e., plasma proteins) or to other tissues in the body is temporally orientated. Metabolism and elimination rates of a drug often are influenced by the time of day at which it enters the system, thereby affecting the relative breakdown of the drug. Furthermore, the combination of these processes may differentially alter how the drug is used by the body (i.e., the bioavailability) depending on the time of administration. However, the bioavailability of alcohol may differ for various target organs. Therefore, a person may be more likely to display the motor effects of alcohol when it is ingested at noon but may display the greatest thermal response during the early morning hours (Brick et al. 1984).

#### Chronesthesy

The second component of efficacy and time of day is chronesthesy. Chronesthesy explains the cyclic “changes in the susceptibility or the sensitivity of a target system” (Reinberg 1992, p. 57). In many ways, chronesthesy is similar to the field of pharmacodynamics, in that it relates to the physiological and biochemical changes associated with a drug depending on the time of administration. Overall, the field of chronesthesy asserts that the performance of a drug often changes as a result of variations that occur on a cellular level. For example, alterations in key proteins on the cell membrane (i.e., receptors on the surface of brain cells) or changes in the way the cell membrane responds to chemicals involved in cellular communication (that is, the degree of membrane permeability) occur on a relatively predictable time scale.

### Chronergy

The second division of chronopharmacology is that of chronergy. This branch of study takes into account not only the influence that time of day may have on a drug’s effect, but also the effect that the drug itself may have on the organism’s biological rhythm. In other words, chronergy synthesizes information obtained from both chronopharmacokinetics and chronesthesy to interpret the influence of a drug on the individual as a whole. This area is far less represented in the existing literature, but has gained considerable attention in recent years and is quickly developing into one of the more popular branches of chronopharmacology.

## Alcohol and Temperature

### Alcohol-Induced T_b_ Changes

Numerous reports in the past have asserted that alcohol lowers T_b_. Initially this response was thought to be caused by a disruption of the cell membranes, which, in turn, caused a change in how easily molecules enter and exit the cell (that is, a change in fluidity). Most studies now suggest, however, that alcohol produces a dysregulation of the thermoregulatory system. This thermodysregulation raises the animal’s temperature to higher than normal (i.e., causes hyperthermia) when the surrounding or ambient temperature (T_a_) is warm and, conversely, decreases the animal’s temperature to lower than normal (i.e., causes hypothermia) when the outside temperature is cooler (Kalant and Le 1991; Alkana et al. 1996). However, when mice are given the opportunity to choose their T_a_, they are known to seek out colder temperatures after alcohol is administered (Huttunen 1990). Similarly, research shows that by concurrently monitoring the selected T_a_ and T_b_, scientists can observe decreases in both of these measures after an animal receives moderate-to-large doses of alcohol (Crawshaw et al. 1998). Together these findings indicate that in addition to impairing thermoregulation, alcohol may alter the set point for T_b_ (Kalant and Le 1991; Crawshaw et al. 1992).

Currently, researchers believe that alcohol exerts its effects both on the brain (i.e., centrally) and on the peripheral body (i.e., peripherally) (Huttunen 1990). Centrally, alcohol-induced thermoregulation, or dysregulation, is thought to occur in a part of the brain responsible for processing incoming sensory signals (i.e., the anterior hypothalamic preoptic area, or AH/POA) (Kalant and Le 1991; Alkana et al. 1996). Almost all nerve-cell-communication chemicals are thought to be involved in alcohol-induced hypothermia (Kalant and Le 1991; Crawshaw et al. 1992). However, the AH/POA shows a particularly increased release of one chemical (i.e., norepinephrine) with the administration of alcohol, which, in turn, appears to cause a decrease in T_b_ (Huttunen 1990). This same release of norepinephrine is observed peripherally and may result from signals (i.e., afferent pathways) to the portion of the brain (i.e., the hypothalamus) that induces production of this hormone.

Scientists also have reported that alcohol-induced hypothermia is correlated with a decreased release of another key chemical involved in nerve-cell communication (i.e., serotonin) (Huttunen 1990; Alkana et al. 1996). Conversely, other studies have associated drops in T_b_ with higher serotonin levels in the brain (Crawshaw et al. 1992). Admittedly, the neurochemical relationship between alcohol and T_b_ needs additional clarification (Kalant and Le 1991; Crawshaw et al. 1992). Nonetheless, these findings suggest that alcohol produces direct alterations on specific nerve cells in the brain that produce T_b_ changes in addition to the widespread cellular effects it produces throughout the central nervous system.

Another portion of the hypothalamus, the suprachiasmatic nucleus (SCN), is the main timekeeping center of the body and is responsible for the development, maintenance, and coordination of bodily CRs. Significant for this discussion, the SCN does not assert a direct role in maintaining normal body function (that is, maintaining homeostasis), such as T_b_. Rather, this nucleus works at a higher level to control the overall generation of the CR, which constantly underlies these important body functions (Refinetti 1995*a*, 1995*b*).

## Temperature-Induced Changes in Alcohol Elimination and Intoxication

T_b_ has been reported as a major factor in the rate in which alcohol is broken down and eliminated from the body (Romm and Collins 1987). Specifically, alcohol-induced hypothermia decreases the rate of chemical reactions, thereby slowing the rate of elimination. Through various metabolic mechanisms, T_b_ has reportedly affected the rate of alcohol elimination by up to 50 to 60 percent (Romm and Collins 1987).

**Figure 2:**
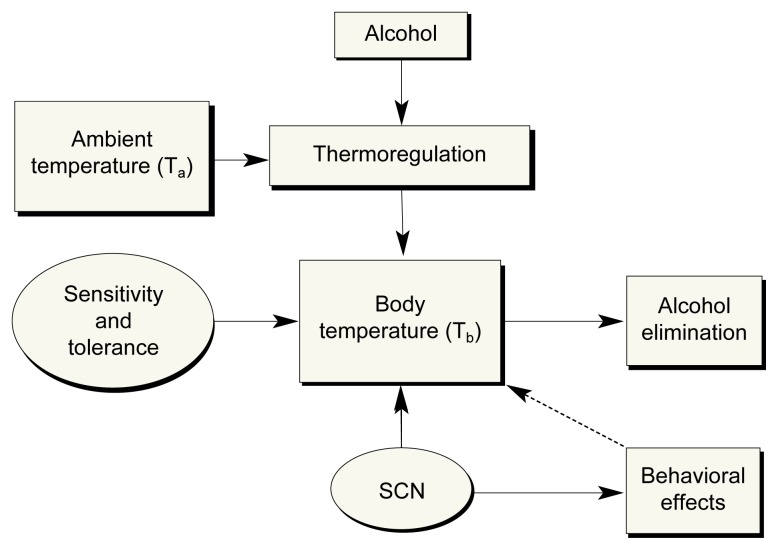
Body temperature (T_b_) can be significantly influenced by environmental or ambient temperature (T_a_) in the presence of alcohol. Alcohol-induced disruption of normal thermoregulatory mechanisms results in hyperthermia or hypothermia in response to higher or lower T_a_, respectively. A lower T_b_ appears to be protective of the system, because hypothermia causes the body to be less sensitive to the central depressant effects of alcohol. Initial sensitivity or tolerance to alcohol also may play a part in the overall behavioral effects. The circadian rhythm influences T_b_ or behavior (e.g., amount of activity) via an area of the brain involved in regulating bodily rhythms (i.e., the suprachiasmatic nucleus [SCN]).

As previously mentioned, alcohol has the ability to induce hypothermia or hyperthermia, depending on the temperature in the environment. Therefore, to include alcohol in the equation, one must consider the influence of T_b_ on the elimination of alcohol (see figure 2). Recent studies have found that behavioral and brain sensitivity to alcohol are indirectly dependent on the environmental temperature (Alkana et al. 1996). Specifically, Alkana’s review discusses a number of studies that describe how increases in external temperature result in hypersensitivity of rodents to alcohol, as indicated by various behavioral measures, including higher levels of mortality. Romm and Collins (1987) hypothesized that after repeated alcohol administration, the behavioral tolerance observed might actually be a development of tolerance to the depressant effects of hypothermia, which, in turn, increases the metabolism of alcohol. Studies such as these reiterate the important influence that alcohol has on thermoregulation and lead to the idea that alcohol-induced hypothermia is a tailored, or adaptive, response most likely found within a variety of organisms (Crawshaw et al. 1992; Alkana et al. 1996).

## Physiological and Behavioral Effects of Alcohol as a Function of Time of Day

### Changes in Sensitivity to Alcohol

Sensitivity to alcohol also is altered according to the time of day that the alcohol is consumed. In rats, sensitivity (as measured by hypothermia) is commonly thought to increase at nighttime (i.e., the dark phase) compared with daytime (i.e., the light phase) (Crawshaw et al. 1992; Brick et al. 1984). The absorption, distribution, and metabolism of alcohol were reported to be unaltered in these studies, implying that the differences which occur result from circadian changes in brain sensitivity and not from changes in the peripheral body (Alkana et al. 1996).

However, other, more straightforward explanations may account for these circadian effects. For instance, the sedative effects of alcohol may cause a decrease in the normally high levels of activity seen in these animals during nighttime (which therefore reduces T_b_), or a “basement” effect of T_b_ may occur, whereby the already low daytime T_b_ cannot be reduced any further. Furthermore, the review by Alkana and colleagues (1996) concluded that when baseline T_b_s were taken into account, the major hypothermic and hyperthermic responses actually were observed during the light-to-dark and dark-to-light transition times, respectively. All of these factors—circadian, peripheral, direct, and indirect—need to be assessed individually to determine the role that time of day plays in sensitivity to alcohol’s effects.

### Alcohol Preference as a Function of Time of Day

As nocturnal animals, many rodents engage in a greater degree of activity during the nighttime than in the daytime. Studies have reported that rats and other rodents prefer alcohol to a greater degree when in a dark environment, in that these animals drink more alcohol during the dark period of an alternating light-dark (i.e., L:D) schedule. Likewise, animals placed in constant dark (i.e., D:D) environments will drink significantly more than animals placed on L:D schedules (Geller and Purdy 1979). It seems likely, then, that a synergistic effect of CR and typical rodent behavior occurs.

The release of the hormone melatonin from the pineal gland appears to play a role in the development of such “dark preferences.” A few observations support this hypothesis. First, animals kept in dark environments reportedly have larger pineal glands, the gland responsible for melatonin production. Second, daily melatonin administration produces a preference for alcohol in animals that previously preferred water. Third, artificially increased levels of serotonin, which is depleted at night because of its conversion to melatonin, decrease an individual’s preference for alcohol. However, the findings surrounding melatonin and serotonin are largely circumstantial and require further investigation to confirm such a relationship (Geller and Purdy 1979).

Shifts in an organism’s normal CR also have been found to induce alcohol consumption (Gauvin et al. 1997*a*). Those shifts include phase delays in which the peak of a body rhythm (such as a peak in temperature) occurs at, or shifts, to a point later in the cycle. Likewise, a phase advance causes a shift in the cycle to an earlier time.

The study by Gauvin and colleagues (1997*a*) found that a single large phase advance produced significant increases in the amount of alcohol consumed by rats for 3 days following the shift, with the greatest increase occurring on the second day. This animal model resembles the various symptoms of jet lag seen in humans, in that it may take 2 to 3 days before the maximal effects of phase shifting are displayed. Conversely, a phase delay of the same magnitude produced only minimal changes in drinking behavior, with increases occurring only on the day of the shift (Gauvin et al. 1997*a*). These findings appear to contradict Geller and Purdy’s findings, in which a greater degree of drinking occurred after increasing the light portion of the cycle on the first day (i.e., phase advance).

Repeated daily shifts in the amount of light and dark (i.e., photoperiod) during a 2-month period (similar to long-term rotating shift work in humans) also produced significant increases in alcohol intake (Gauvin et al. 1997*a*). Gauvin and colleagues have suggested that these photoperiod shifts serve as stressors, which may result in alcohol use, possibly as a means of self-medicating the desynchronosis or rhythm disruption.

## Influence of Alcohol on the CR of Body Temperature

Investigators have often stated that in most situations alcohol produces “rebound” hyperthermia, a response by the body to counter the hypothermia initially produced by alcohol. This interpretation, however, was challenged by Gallaher and Egner (1987), who proposed that in certain situations the hyperthermia observed may not be a homeostatic rebound effect, but rather “an abolition of the normal circadian temperature rhythm” (p. 38). Supporting this proposal, Gauvin and colleagues (1993) found that the “hyperthermia” exhibited by animals after a bout of alcohol-induced hypothermia did not exceed normal circadian fluctuations. Therefore, alcohol appears to shift the circadian T_b_ cycle, but not the absolute magnitude of T_b_. In other words, the delayed hyperthermic rebound effect is not a physiological response at all, but rather an effect that results from the absence of the normal low in the circadian rhythm of T_b_ (Holloway et al. 1993).

The involvement of SCN in T_b_ regulation was touched on in an earlier section of this article. Research has suggested that although this section of the brain does not respond directly to individual homeostatic fluctuations of T_b_, it does act as an overall regulator, ensuring that the organism can adapt to fluctuations in the environmental cycle. This interpretation does not necessarily provide support for the interconnection between alcohol and CR through direct mechanisms. Instead, indirect modulation, by way of alcohol’s disruptive effects on the hormonal and chemical communication networks involved in maintaining the body’s temperature balance, also must be considered in studies such as the following.

Few experiments have taken a systematic approach toward elucidating alcohol’s direct effects on CR. Recently, however, Baird and colleagues (1998) examined the effects produced when alcohol was administered at various times throughout the day. Under a 12:12 L:D cycle (that is, 12 hours of daylight and 12 hours of nighttime, with lights on at 6:00 a.m.), a number of time- and dose-dependent alterations in circadian T_b_ parameters were demonstrated.

First, alcohol-treated animals showed significantly shorter (i.e., less than 24 hours) T_b_ rhythm periods or cycles. Baird and colleagues (1998) suggested that this patterning may signify that CR is susceptible to initial temperature-lowering effects of alcohol (i.e., indirect pathways) or that alcohol is able to alter the length of the period of the T_b_ rhythm directly.

Second, animals exhibited a dose-dependent decrease in T_b_ rhythm (i.e., amplitude) when alcohol was administered at 7:00 p.m. The hypothermic response of alcohol administered at this time also might be countered by the hyperthermic rebound effect brought on by the animal’s increased nighttime activity. Although the assertion by Baird and colleagues requires further support, they suggested that their findings endorse the Gallaher and Egner theory presented previously, which interprets this change in temperature as a phase shift in the T_b_ rhythm rather than as a homeostatic response to the alcohol-induced hypothermia.

Third, several acrophase (i.e., the time it takes to reach the peak T_b_ of the rhythm from an arbitrary reference point, usually midnight) changes were uncovered in response to alcohol administration. The study conducted by Baird and colleagues (1998) found that phase delays were produced when alcohol was administered at 11:00 p.m. Those delays were believed to result from the initial hypothermia typically induced by alcohol—that is, alcohol-induced hypothermia prevented the normal cyclic increase in T_b_ observed at that time. However, when alcohol was administered at 1:00 a.m., phase advances were observed. Those advances cannot be explained in the same way as the phase delays—that is, as an indirect result of alcohol-induced hypothermia. This finding further strengthens the theory that alcohol acts directly on the central pacemaker. Baird and colleagues proposed that alcohol alters the overt T_b_ rhythm, not the peripheral system, which, in turn, might alter the SCN.

It should be taken into account, however, that to separate phase advances or delays from the acute effects of alcohol, the shift must be enduring. In an L:D paradigm, such as the one used by Baird and colleagues, the animals were being reconditioned to the light each day. To disentangle these circadian effects, we have conducted a followup study replicating this systematic approach under a D:D schedule. Preliminary results appear to support a similar phase-response curve in free-running, dark-adapted animals.

## SUMMARY

The areas of chronopharmacokinetics and chronopharmacodynamics are now stable pillars in the foundation of alcohol chronopharmacology. Furthermore, in the area of chronergy, it is becoming clearer that alcohol sensitivity and preference vary with circadian timing. However, studies supporting alcohol’s ability to influence the circadian phase are limited.

Our laboratory is continuing to examine the interaction of alcohol and CRs, including the attenuation of the circadian phase shifts engendered by alcohol. In addition to the acute alcohol exposure studies, we are investigating alcohol’s ability to serve as an entrainer of the circadian T_b_ rhythm. This finding would support a direct relationship with the circadian system. Elucidating this relationship will provide the basis for developing techniques (e.g., bright light therapy) and medications (e.g., melatonin) to prevent or treat alcohol abuse and addiction as well as the performance deficits associated with alcohol-induced CR disruption.

Because of the differences between species that are active at night, such as rodents, and species that are active during the day, such as humans, it is difficult to speculate how these rodent models will translate for human application. Recent technological advances in human-circadian-monitoring devices, which can be worn on the wrist much like a watch, may soon clarify this point. We hope that such investigations, along with the rapidly growing body of research presented, will provide a clearer understanding of the unique interplay between alcohol and CR.
